# Visual outcome of various dose of glucocorticoids treatment in nonarteritic anterior ischemic optic neuropathy– a retrospective analysis

**DOI:** 10.1186/s12886-024-03354-4

**Published:** 2024-03-04

**Authors:** Fang-Fang Zhao, Yun Chen, Tai-Ping Li, Yun Wang, Hong-Jie Lin, Jian-Feng Yang, Lan Chen, Shao-Ying Tan, Jia-Jian Liang, Ling-Ping Cen

**Affiliations:** 1 Joint Shantou International Eye Center of Shantou University and The Chinese University of Hong Kong, North Dongxia Road, 515041 Shantou, Guangdong China; 2 Shantou University Medical College, Shantou, Guangdong China; 3 The Fourth Hospital of Wuhan, Wuhan, China; 4 School of Optometry, The Hong Kong Polytechnic University, Kowloon, Hong Kong China

**Keywords:** Acute nonarteritic anterior ischemic optic neuropathy (NAION), Glucocorticoid therapy, Best-corrected visual acuity (BCVA), Dosage comparison

## Abstract

**Background and purpose:**

The objective of this investigation was to assess the therapeutic efficacy of distinct glucocorticoid therapy dosages in the management of acute nonarteritic anterior ischemic optic neuropathy (NAION).

**Materials and methods:**

This retrospective, unmasked, and non-randomized study included a total of 85 patients. The patients were categorized into four groups: Group 1 (control) consisted of 15 patients who did not receive glucocorticoids, Group 2 included 16 patients administered with oral prednisone at a dosage of 1 mg/kg/d for 14 days, Group 3 comprised 30 patients who received 250 units of methylprednisolone once daily for 3 days, followed by oral prednisone at a dosage of 1 mg/kg/d for 11 days, and Group 4 encompassed 24 patients who received 500 units of methylprednisolone once daily for 3 days, followed by oral prednisone at a dosage of 1 mg/kg/d for 11 days. The best-corrected visual acuity (BCVA) was assessed at baseline and the final follow-up (> 7 days post-treatment). The changes in visual acuity between baseline and the 7–14 day follow-up, as well as between baseline and the concluding appraisal, were employed as metrics for assessing the extent of visual enhancement.

**Results:**

No significant differences were noted in the final visual outcomes or in the changes between final visual acuity and baseline across the four groups. In Group 1 (control), the best-corrected visual acuity (BCVA) remained unchanged during final follow-ups compared to baseline. Conversely, the intervention groups exhibited statistically significant enhancements in BCVA during final follow-up (*p* = 0.012, *p* = 0.03, and *p* = 0.009 for Group 2, Group 3, and Group 4, respectively) when compared to baseline. During the 7–14 day follow-up, there was a significant difference in the changes between baseline BCVA and follow-up BCVA across the groups (*p* = 0.035). Go a step further by Bonferroni correction for multiple comparisons, group 4 showed a greater change in vision compared with group1 (*p* = 0.045).

**Conclusion:**

Our study on acute nonarteritic anterior ischemic optic neuropathy (NAION) showed no significant final visual outcome differences. Nevertheless, Groups 2, 3, and 4 demonstrated improved best-corrected visual acuity (BCVA) during the final follow-up. Notably, a 500-unit dose of methylprednisolone resulted in short-term BCVA enhancement. This suggests potential consideration of 500 units of methylprednisolone for short-term NAION vision improvement, despite its limited long-term impact.

## Introduction

Anterior optic neuropathy arises from diminished blood supply to the optic nerve head and is categorized into arteritic anterior ischemic optic neuropathy (A-AION) and non-arteritic anterior ischemic optic neuropathy (NAION). NAION stands as the predominant cause of optic nerve swelling and optic neuropathy in adults aged over 50 years [1], with an incidence rate of 2.5–11.8 per 100,000 cases among men over 50 [2]. NAION manifests as an abrupt, painless decline in unilateral vision, primarily affecting the upper and lower visual field halves, commonly upon awakening [3–5]. Conventional approaches to risk mitigation encompass the management of factors such as diabetes, hypertension, hyperlipidemia, and sleep apnea.

While the precise pathogenesis of NAION remains elusive, the predominant hypothesis implicates hypoperfusion and ischemia of the short posterior ciliary arteries that supply the optic nerve. This process results in optic nerve edema, subsequently triggering ischemia, which leads to swelling of the optic nerve segment traversing a small aperture within the scleral canal [6]. Consequently, this optic nerve edema induces a compartment syndrome involving neighboring axons compressed within the confined space of the scleral canal opening. The outcome is apoptosis and degeneration of ganglion cells, the axons of which form the optic nerve [7]. A proposed mechanism suggests that glucocorticoids could mitigate capillary permeability, expedite the resolution of disc edema, alleviate capillary compression around the optic nerve head, and thereby enhance blood flow to ischemic axons [8, 9]. Differences exist in doses and effects of various glucocorticoids administered in prior studies. In this study, we explore the influence of diverse glucocorticoid therapy dosages on NAION, with a specific emphasis on visual outcomes.

## Materials and methods

### Study cohort

This retrospective, unmasked, and non-randomized study was conducted at the Joint Shantou International Eye Center of Shantou University and The Chinese University of Hong Kong between 2012 and 2021.

The primary assessment parameter was best-corrected visual acuity (BCVA), and the changes in visual acuity between baseline and the 7–14 day follow-up, as well as between baseline and the final assessment, were measured. The objective of the trial was to evaluate whether glucocorticoid therapy improves visual outcomes and to determine the most effective dosage for the final follow-up after a minimum treatment duration of 7 days.

The diagnostic and inclusion criteria for patients with NAION are as follows: (1) sudden loss of vision associated with relative afferent pupillary defect within 2 weeks of onset, (2) sector or diffuse optic disc edema, (3) field defects corresponding to disc change, and (4) negative erythrocyte sedimentation rate (ESR) and C-reactive protein (CPR) to exclude A-AION. Brain or orbital Magnetic Resonance Imaging (MRI) and infection-related laboratory tests were performed in case of other suspected optic neuropathy diseases. Moreover, having a disc-at-risk in the fellow eye with a cup-to-disc ratio of 0.2 or less was considered a secondary diagnostic index [7]. Exclusion criteria for the study were: (1) previously documented retinal conditions that could influence VA, such as severe non-proliferative or proliferative diabetic retinopathy. Patients with mild non-proliferative diabetic retinopathy without macular edema were included; (2) glaucoma, or any other ocular diseases that could influence VA; (3) discontinuation of treatment due to glucocorticoid complications.

For analysis, patients were retrospectively allocated to four groups: group 1 (control) comprised 15 patients without glucocorticoid treatment, group 2 included 16 patients administered oral prednisone at 1 mg/kg/d for 14 days, group 3 involved 30 patients receiving 250 units of methylprednisolone once daily for 3 days, followed by oral prednisone at 1 mg/kg/d for 11 days, and group 4 encompassed 24 patients who received 500 units of methylprednisolone once daily for 3 days, followed by oral prednisone at 1 mg/kg/d for 11 days. Chest radiographs and electrocardiograms were conducted for the intervention groups. A team of proficient physicians managed potential glucocorticoid-related side effects throughout the entire process.

### Study protocol

All participants underwent a comprehensive ophthalmic assessment, encompassing Snellen best-corrected visual acuity (BCVA) measurement, slit-lamp examination, intraocular pressure (IOP) measurement, Humphrey automated static perimetry for visual field assessment (VF), and dilated fundus examination. Visual parameters, converted to logarithm of the minimum angle of resolution (logMAR) units for statistical analysis, were documented at baseline and the final follow-up. For patients experiencing successive bilateral onset, record the eye affected initially.

### Statistical methods

Statistical analysis was carried out using SPSS software version 25.0 (IBM Co., Chicago IL). Quantitative data were expressed as mean ± standard deviation or median and range, as appropriate. Appropriate parametric and nonparametric tests, including the chi-square test, analysis of variance (ANOVA), the paired Student’s t-test, Mann-Whitney U Test and Kruskal-Wallis test, were applied reasonably. Bonferroni-corrected pairwise comparisons were used to adjust for multiple comparisons. P values less than 0.05 were taken as statistically significant.

## Results

### Patients’ demographics

The main demographic and clinical data of the study are depicted in Table 1. Four groups were statistically similar regarding age, gender, time of onset, short-term follow-up time, and the final follow-up time point. The mean ages of the patients were 56.5, 58.8, 57.9, and 53.0 years, respectively (*p* = 0.203, Table 1), and female preponderance was observed in all four groups. The median time from onset was 10.0 days (range, 7.0–14.0 days), 12.5 days (range, 7.0–14.0 days), 10.0 days (range, 7.0–10.0 days), and 10.0 days (range, 9.3–14.0 days) in group 1, group 2, group 3, and group 4, respectively (*p* = 0.335; Table 1). The corresponding values at 7–14 day follow-up time point were 14.0 days (range, 7.0–14.0 days), 8 days (range, 7.0-11.8 days), 8.0 days (range, 7.0–9.0 days), and 9.0 days (range, 7.0–11.0 days) (*p* = 0.102; Table 1); and corresponding final follow-up point were 41.0 days (range, 14.0-208.0 days), 25.5 days (range, 14.8–623.0 days), 8.0 days (range, 13.0-149.5 days), and 60.0 days (range, 22.8–318.0 days), respectively (*p* = 0.252; Table 1).

**Fig. 1:**
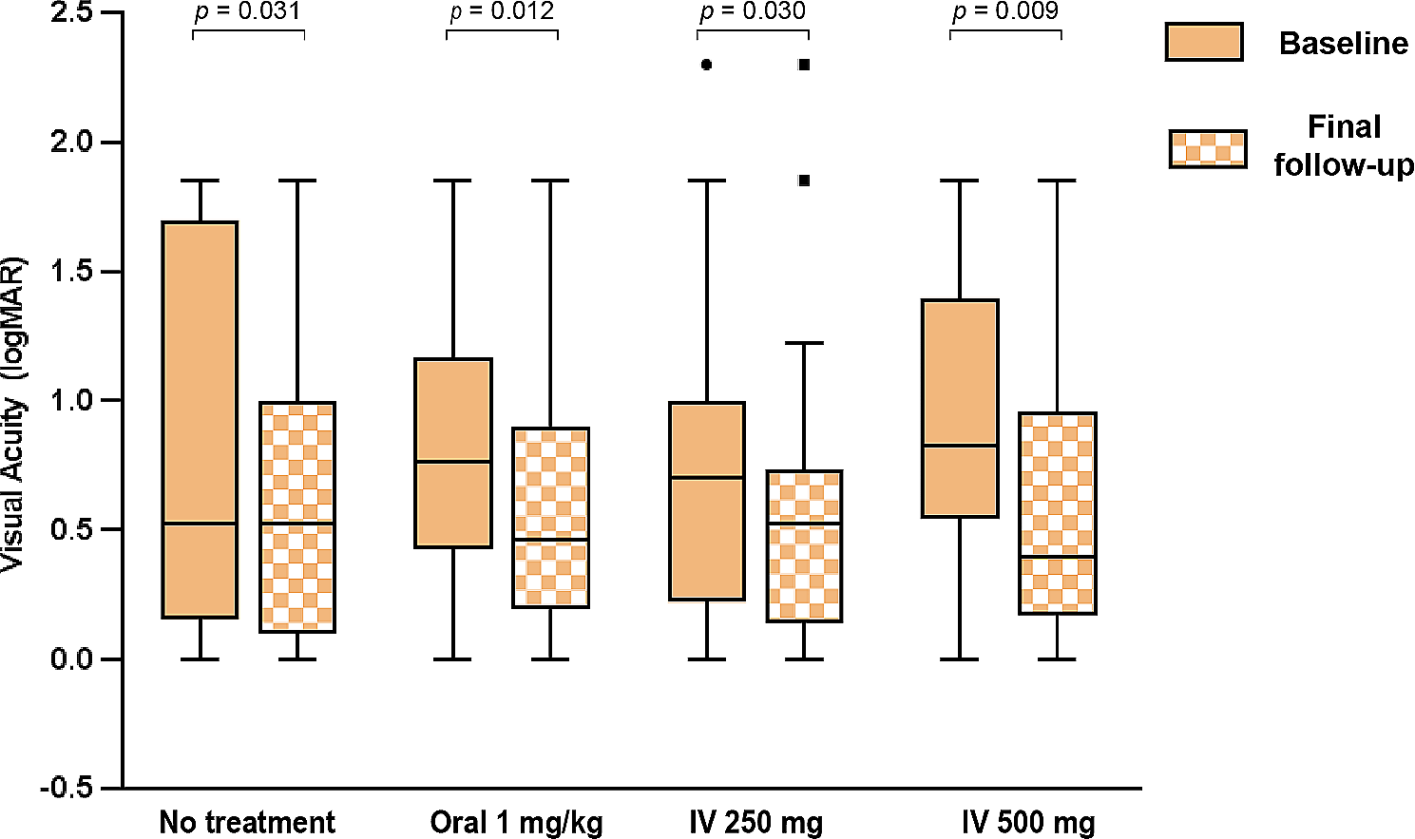
Boxplot illustrating BCVA at baseline and during the final follow-up. The diagram presents the median, along with the first and third quartile values

**Fig. 2:**
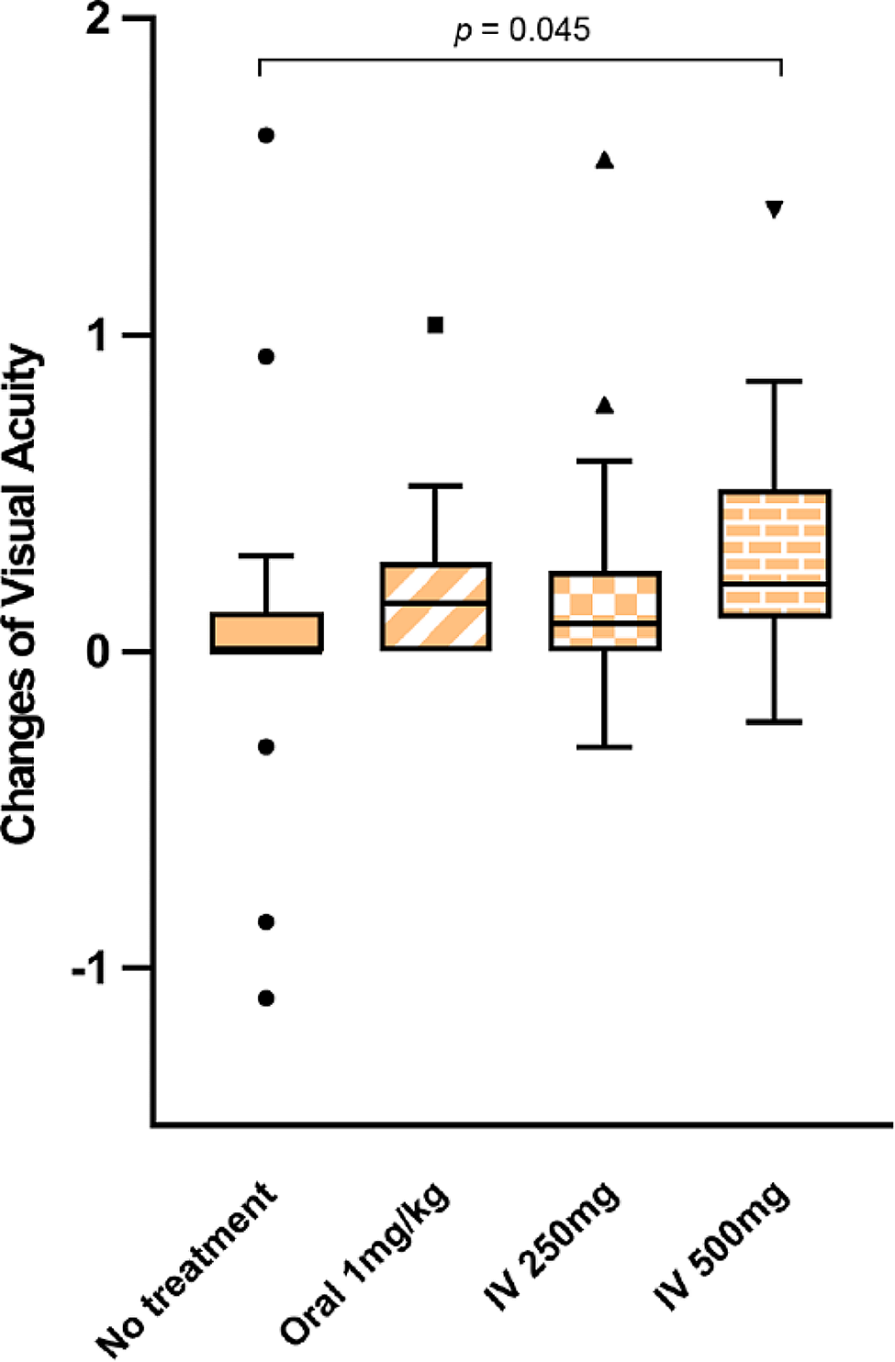
Boxplot depicting visual acuity changes between baseline and short-term follow-up in the 4 groups. *P* value for the difference between group 1 and group 4 is 0.045 by Bonferroni correction for multiple comparisons

The median baseline BCVA was 0.52 logMAR (range, 0.15–1.70 logMAR), 0.76 logMAR (range, 0.43–1.17 logMAR), 0.70 logMAR (range, 0.22-1.00 logMAR) and 0.82 logMAR (range, 0.54–1.40 logMAR), respectively (*p* = 0.495; Table 2). At final follow-up time, the corresponding values were 0.52 logMAR (range, 0.97-1.00 logMAR), 0.46 logMAR (range, 0.19–0.90 logMAR), 0.52 logMAR (range, 0.14–0.73 logMAR) and 0.40 logMAR (range, 0.17–0.96 logMAR), respectively (*p* = 0.974; Table 2). There was no difference in final visual outcome (Table 2; Fig. 1) between the four groups. In group 1 (control) BCVA remained the same at final follow-up time with the baseline. On the contrary, intervention groups showed a statistical visual improvement from baseline to the final follow-up (*p* = 0.012, *p* = 0.03, and *p* = 0.009 for group 2, group 3 and group 4, respectively) (Table 2; Fig. 1). It did not show a greater change in vision between groups at final follow-up time (Table 2), but at 7–14 day follow-up (*p* = 0.035, Table 2). Go a step further by Bonferroni correction for multiple comparisons, group 4 showed a greater change in vision compared with group 1 (*p* = 0.045, Fig. 2). No serious adverse effects of steroid therapy were documented in this study.

**Table 1:** Clinical and demographic profile of patents, including time from onset, 7–14 day follow-up and final follow-up for each of the groups

	Total	Groups				P value
		Group1	Group2	Group3	Group4	
Age (yrs), mean ± SD	56.4 ± 9.7	56.5 ± 11.7	58.8 ± 8.2	57.9 ± 8.5	53.0 ± 10.2	0.203#
Gender (male:female)	29/53	4/11	5/9	12/18	8/15	0.836$
Time from onset, median (range day)	10 (7.0–14.0)	10 (7.0–14.0)	12.5 (7.0–14.0)	10 (7.0–10.0)	10 (9.3–14.0)	0.335*
Short-term follow-up (day)	9 (7.0–11.0)	14 (7.0–14.0)	8 (7.0–11.8)	8 (7.0–9.0)	9 (7.0–11.0)	0.102*
Final follow-up (day)	43 (14.5-214.5)	41 (14.0-208.0)	25.5 (14.8–623.0)	27 (13.0-149.5)	60 (22.8–318.0)	0.252*

#Based on Chi-Square test

$Based on ANOVA

*Based on Kruskal-Walls test

**Table 2:** Best-corrected visual acuity (BCVA) during the course of the study for each of the groups

	Time		Total	Groups				P value
				Group1	Group2	Group3	Group4	
BCVA	Baseline	Value	0.70 (0.28–1.22)	0.52 (0.15–1.70)	0.76 (0.43–1.17)	0.7 (0.22–1.00)	0.82 (0.54–1.40)	0.495^*^
	7–14 day follow-up (day)	Value	0.40 (0.22–0.82)	0.52 (0.97–1.40)	0.61 (0.24–0.96)	0.40 (0.19–0.82)	0.35 (0.30–1.07)	0.936^*^
		Change	0.125 (0.000–0.398)	0.000 (0.000–0.125)	0.102 (0.000–0.242)	0.088 (0.000–0.253)	0.213 (0.104–0.511)	0.035^*^
		P-within		0.635	0.008	0.009	< 0.001	
	Final follow-up (day)	Value	0.46 (0.15–0.82)	0.52 (0.97–1.00)	0.46 (0.19–0.90)	0.52 (0.14–0.73)	0.4 (0.17–0.96)	0.974^*^
		Change	0.125 (0.000–0.398)	0.058 (−0.058 to 0.301)	0.161 (0.836–0.281)	0.088 (−0.097 to 0.398)	0.349 (0.000–0.595)	0.277^*^
		P-within		0.31^#^	0.012^†^	0.03^#^	0.009^#^	

*Based on Kruskal-Wallis test

†Based on paired student’s t-test

#Based on Mann-Whitney U Test

## Discussion

Our retrospective study investigated the therapeutic efficacy of distinct glucocorticoid therapy dosages in NAION. While no significant differences were observed in the final visual outcomes across the studied groups, the intervention groups (Groups 2, 3, and 4) demonstrated statistically significant enhancements in BCVA during the final follow-up compared to baseline.

Based on previous research, if the course of the disease is within 2 weeks, glucocorticoid treatment can significantly improve vision and visual field, and the absorption of disc edema can also be significantly accelerated [10–15]. Hayreh [11] demonstrated a better vision outcome after administration of oral steroids compared to the untreated group at 6 months, but the results were opposite to the later studies where various doses of glucocorticoids, including oral prednisone, 500 units of methylprednisolone, and 1000 units of methylprednisolone, were used to treat NAION patients [16–21] (Table 3). In our study, we administered treatment to NAION patients within two weeks of symptom onset, employing varying glucocorticoid dosages. Although the intervention groups exhibited statistically significant improvements in BCVA from baseline to final follow-up evaluations, we observed no notable differences in short-term or final visual outcomes across the four groups. Our results are consistent with previous investigations [16–21], diverging from the findings of Hayreh’s study [11].

Notably, during the 7–14 days follow-up period, patients receiving 500 units of methylprednisolone exhibited a significantly greater improvement in BCVA, suggesting a short-term benefit for vision enhancement in our retrospective study. Saxena [16] disclosed that a significant greater change in BCVA was noted in the oral prednisone group compared with the nonsteroid group. In our study, patients who received 500 mg of methylprednisolone, but not oral prednisone, exhibited a more favorable change than the control patients. Discrepancies between the two studies could be attributed to variations in prednisone dosage and treatment duration. Saxena administered prednisone at 80 mg for 2 weeks, followed by a tapering regimen to 70 mg for 5 days, 60 mg for 5 days, and subsequent reductions of 5 mg every 5 days until cessation. In contrast, our study employed oral prednisone at a dosage of 1 mg/kg/d for 14 days. Despite the differences between the two investigations, the results suggest a potential advantageous effect of steroid utilization.

Efforts to delve deeper into the pathogenesis of NAION warrant further attention. While a hypothesized cycle of disc edema induced by compartment syndrome within a confined space has been proposed [22], the extent to which angiogenesis or cytotoxicity may also contribute to optic disc edema remains unexplored. Furthermore, early stages of white matter (optic nerve) infarction are characterized by substantial cellular inflammation, involving the participation of polymorphonuclear leukocytes and macrophages in debris clearance and tissue restructuring. Analogous to ischemic strokes in the central nervous system, pure axonal ischemia leads to the prompt recruitment of extrinsic macrophages to the ischemic area. The optic nerve in both NAION and its primate model exhibits early cellular inflammation, potentially contributing to postinfarct optic nerve damage [23, 24]. Glucocorticoids, with a broad spectrum of activities including anti-inflammatory, anti-angiogenic, and anti-edema, have been the clinical mainstay for the management of perilesional vasogenic edema [25–27].

Although the precise mechanism of action underlying glucocorticoid treatment remains uncertain, it appears that employing glucocorticoid therapy to modulate the inflammatory response and alleviate optic disc edema could potentially serve as a logical factor for improving visual outcomes in individuals with NAION.

## Conclusion

Unfortunately, due to the retrospective nature of this study, our statistical conclusions could not be supported by the most compelling evidence. Nonetheless, our study has effectively assessed the efficacy of glucocorticoid therapy in patients with NAION across various dosages. Our findings reveal that during the 7–14 days follow-up period, patients who received 500 units of methylprednisolone exhibited a notably greater disparity in BCVA compared to the baseline. As a result, for patients aiming to enhance their vision in the short term and lacking contraindications to glucocorticoid therapy, deliberation could be given to the administration of 500 units of methylprednisolone. Nevertheless, it is crucial to concurrently inform patients that the treatment does not influence final visual outcomes. Subsequent research is essential to further optimize treatment strategies.

**Table 3:** Treatment with different doses of glucocorticoids

Glucocorticoid therapy dosage	Author	Publication year	Literature type
Prednisolone: 80 mg for 2 weeks, then tapered down every 5 days	Hayreh [11]	2008	NRCT
	Saxena [16]	2018	RCT
	Rebolleda [18]	2013	NRCT
Prednisolone 75 mg daily tapered off in 6 weeks.	Nikkkhah [21]	2020	RCT
Methylprednisolone IV: 1000 mg daily for 3 days, then oral prednisone acetate 1 mg/kg daily for 11 days, with specific tapering	Kinori [17]	2014	NRCT
Methylprednisolone IV: 500 mg twice daily for 3 days, then oral prednisone acetate 1 mg/kg daily for 2 weeks	Pakravan [19]	2016	RCT
Methylprednisolone IV: 500 mg daily for 3 days, then oral prednisone acetate 1 mg/kg daily for 10 days	Pakravan [20]	2017	NRCT

RCT: Randomized Controlled Trial; NRCT: Non-Randomized Controlled Trial

## Data Availability

The datasets analyzed during the current study are available from the corresponding author upon reasonable request.
